# The Solitary Variant of Mandibular Intraosseous Neurofibroma: Report of a Rare Entity

**DOI:** 10.1155/2015/520261

**Published:** 2015-12-06

**Authors:** Pavan Kumar Gujjar, Jayadev M. Hallur, Shruthi T. Patil, Shylaja Mysore Dakshinamurthy, Mayura Chande, Treville Pereira, Jyoti Zingade

**Affiliations:** ^1^Department of Oral and Maxillofacial Pathology, Narsinhbhai Patel Dental College and Hospital, Visnagar, Gujarat 384315, India; ^2^Department of Oral Medicine and Radiology, Narsinhbhai Patel Dental College and Hospital, Visnagar, Gujarat 384315, India; ^3^Department of Oral and Maxillofacial Pathology, D. Y. Patil School of Dentistry, Nerul, Navi Mumbai 400706, India

## Abstract

Neurofibroma (NF) is a benign neoplasm derived from peripheral nerve cells. NF may extend either as a solitary lesion or as part of a generalized syndrome of neurofibromatosis. Intraorally, the intraosseous variant of neurofibroma is a very rare tumor. The literature provides only few cases of solitary intraosseous neurofibroma of the mandible. We report a case of 28-year-old female who was diagnosed with a solitary intraosseous neurofibroma involving the lower left quadrant of the mandible. The present case is rare in regard to its dimensions and its location.

## 1. Introduction

Neurofibroma (NF) is a benign tumor of neuronal origin that occurs as a single or multiple lesion associated with neurofibromatosis type 1 (NF1), which is a systemic condition caused by a germline mutation in the NF1 gene, a tumor suppressor gene located at 17q11.2 [1]. Bruce first described this term of solitary NF of oral cavity in 1954. Very few cases of solitary NF of the oral cavity have been reported in the literature [2]. WHO defines “neurofibroma as a benign tumor of the peripheral nerve sheath phenotype with mixed cellular components which includes Schwann cells, perineural hybrid cells, and intraneural fibroblasts” [3]. Only 6% of NFs occur in the oral cavity [4]. The tongue and buccal mucosa are the most common intraoral sites whereas intraosseous NFs of the mandible are very rare [5]. Here we report a case of solitary intraosseous neurofibroma of the mandible in a young female.

## 2. Case Report

**Figure 1:**
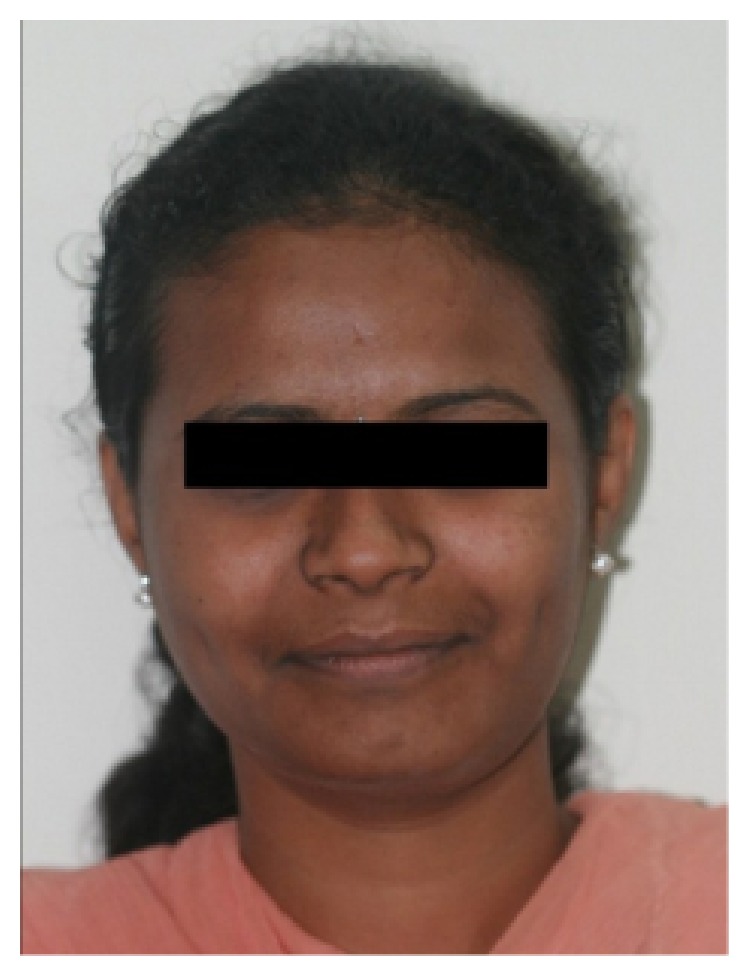
Extraoral photograph showing bilateral asymmetry of the face.

**Figure 2:**
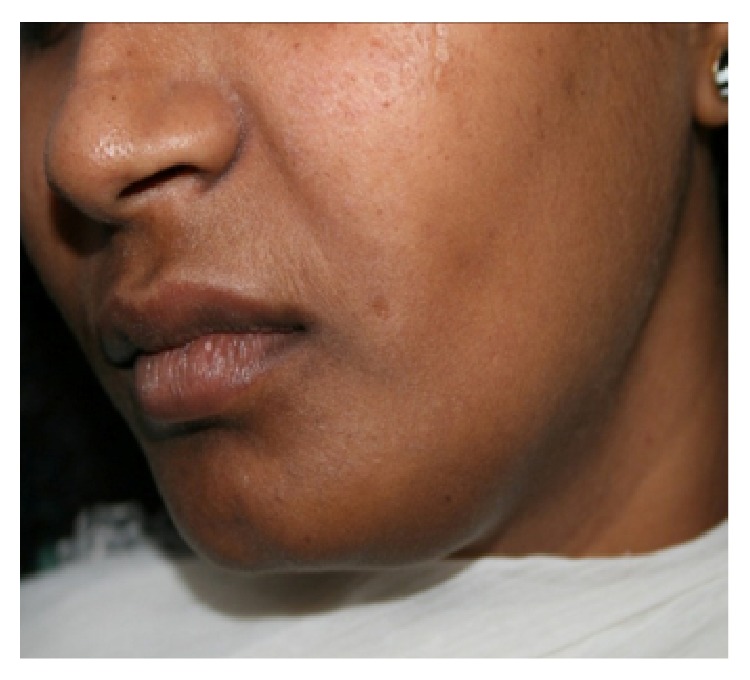
Photomicrograph showing diffuse swelling on lower left side of face.

A 28-year-old female patient visited the Department of Oral Medicine and Radiology, with a chief complaint of swelling in the lower left side of the face (Figure 1). The patient gave history of a slow growing swelling for 3 years with intermittent dull aching pain for the past 3 months. The swelling was initially small and attained its present size gradually (Figure 2). Intraorally there was diffuse swelling extending from 33- to 37-tooth region measuring about 3 × 4 cms in size, oval in shape, and the mucosa overlying the swelling was normal.

**Figure 3:**
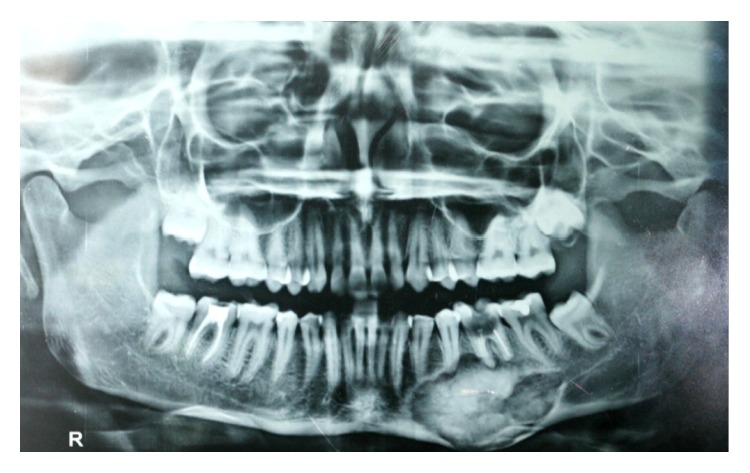
Panoramic radiography showing a homogenous radioopacity with thin radiolucent border from lower left canine to the lower left second molar.

**Figure 4:**
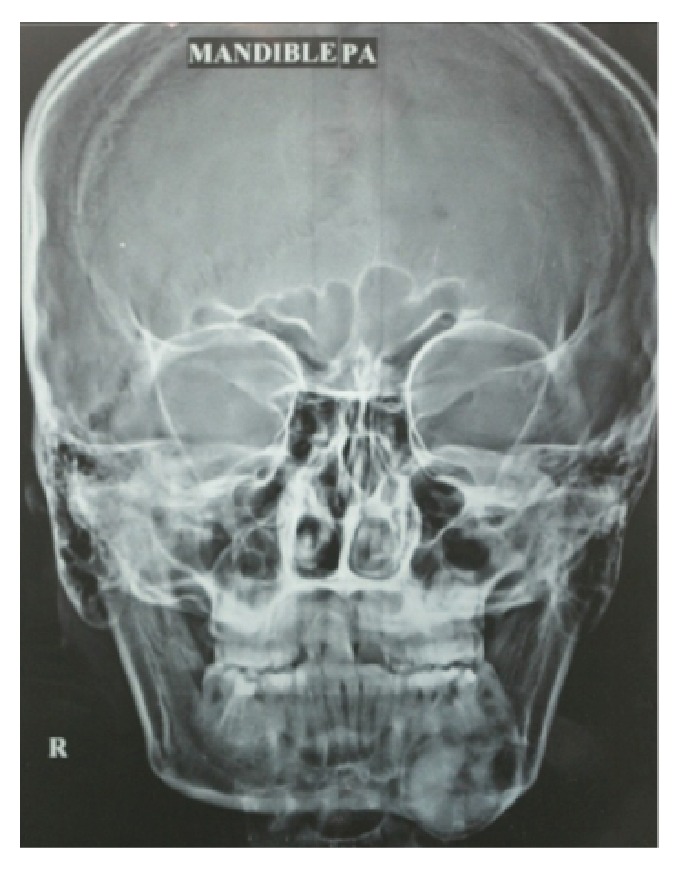
Posteroanterior radiograph showing homogenous radioopacity with thin radiolucent border in the left mandibular posterior region.

On panoramic radiography and posterior-anterior view a homogenous radioopacity surrounded by a thin uniform radiolucent border from 33 to 37 involving the mandibular canal, with no resorption of the roots, was seen (Figures 3 and 4).

**Figure 5:**
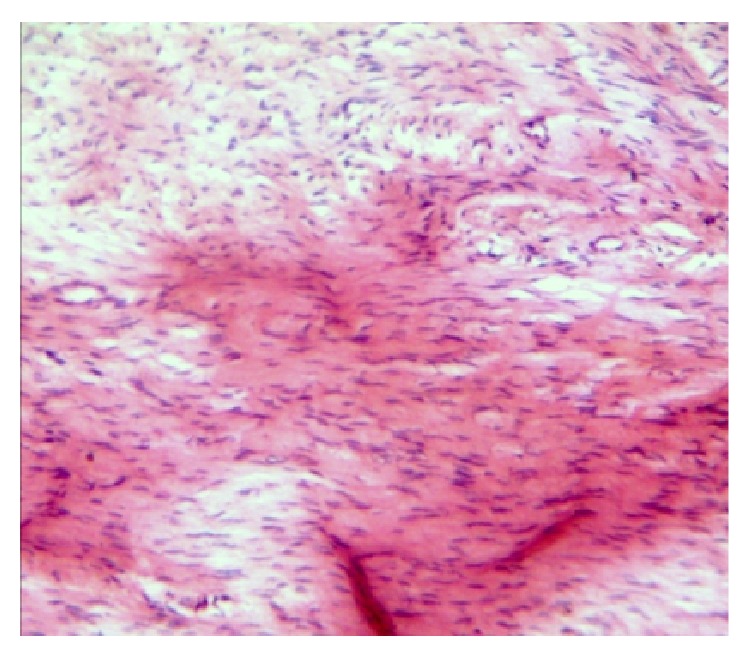
Haematoxylin and eosin stain (40x) showing spindle shaped cells with wavy nuclei and collagen fibres within a myxoid stroma.

Incisional biopsy was done under local anaesthesia, and the specimen was submitted for histopathological examination. The haematoxylin and eosin stained section revealed a benign proliferation of spindle shaped cells with wavy nuclei and collagen fibres within the myxoid stroma. A few mast cells and chronic inflammatory cells' infiltration was observed (Figure 5).

**Figure 6:**
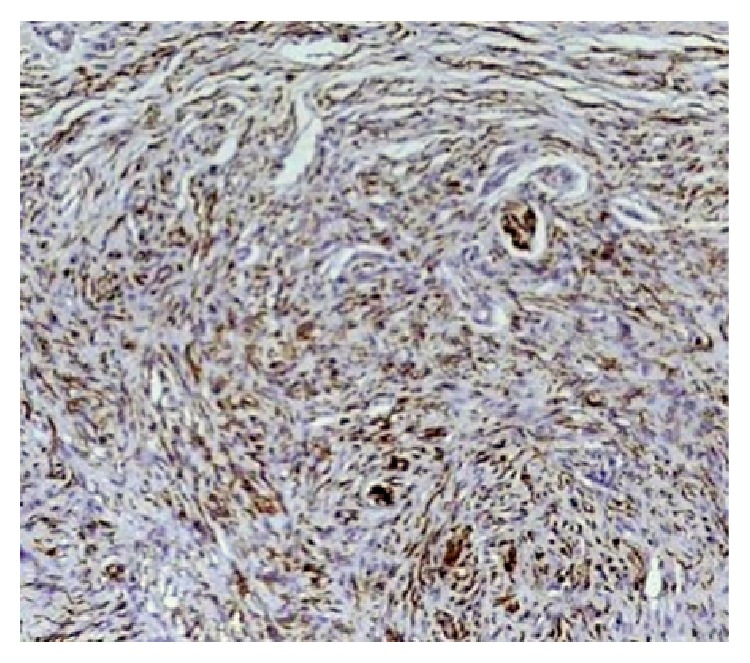
S-100 positivity for nerve tissue and spindle cells (40x).

A diagnosis of neurofibroma was arrived at and immunohistochemical staining using S-100 was done which showed positivity for nerve tissue which confirmed the diagnosis (Figure 6).

**Figure 7:**
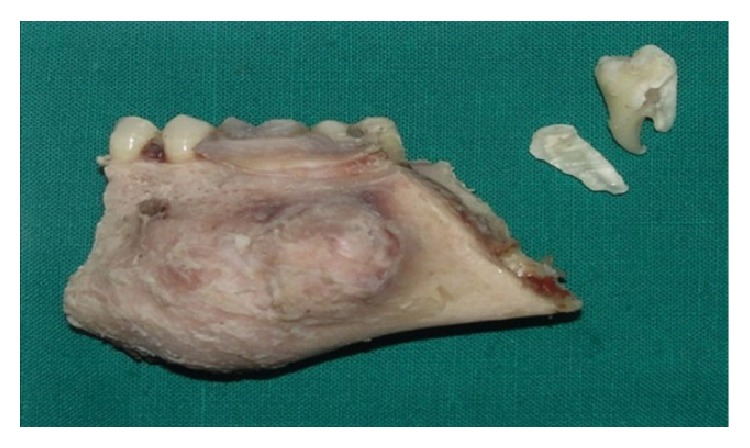
Excised specimen after segmental resection.

Thus, correlating the histopathological and immunohistochemical results, we arrived at a confirmatory diagnosis of neurofibroma. After confirmatory diagnosis a segmental resection of left side of the mandible was performed under general anaesthesia (Figure 7). A clinical follow-up was performed for one year and there was no indication of recurrence.

## 3. Discussion

Neurofibroma (NF) is an uncommon benign tumor of the oral cavity which originates from the cells that constitute the nerve sheath [4]. In the oral cavity NFs often involve the trigeminal and upper cervical nerves. The head and neck are commonly involved because of rich innervations in this area. Superficial involvement of soft tissue is more frequent than the deeper location. In the oral cavity NF is reported to occur on the tongue, lip, palate, gingiva, major salivary glands, and the jaw bones [6].

Approximately 25% of the NFs are seen in the head and neck region, and 5.6% of them occur in the oral cavity. The average age for NFs is 27.5 years and females are more commonly affected [5]. Literature search of intraosseous NFs of the jaws showed few reported cases. Che et al. noticed more number of NFs occurring in the posterior part of the mandible, which could be the reason for passing of bundles of inferior alveolar nerve in the mandibular canal [7].

Most of the intraosseous NFs are asymptomatic in the initial stages. As the tumor increases in its size, it starts compressing on the adjacent vital structures and begins to destroy the bone. Later on pain and numbness of the affected side of the lip occur. So far, few cases of symptomatic intraosseous neurofibroma have been reported [5]. Larsson et al. and Apostolidis et al. reported cases of intraosseous NFs of mandible with symptoms like swelling, pain, paraesthesia, and bone destructions [8, 9]. Vivek et al. reported a case of solitary intraosseous NF of mandible with loosening of teeth on the affected side [10]. Similar to the symptoms reported in the literature search, the present case also depicted the symptoms of swelling, pain, and loosening of teeth (at the time of resection). Histopathologically, NFs exhibit an irregular pattern with interlacing bundles of spindle shaped cells with round or fusiform nuclei, and eosinophilic cytoplasm within a loose matrix of fine fibrillar collagen. It is unencapsulated and composed of a mixture of Schwann, perineural-like, and fibroblastic cells [4]. NFs are immunopositive for S-100 protein, indicating its neural origin. Histopathological analysis supported by immunohistochemistry is essential for the correct diagnosis of these oral soft tissue growths [11, 12].

Spindle cell lesions like schwannoma, traumatic neuroma, spindle carcinoma, and amelanotic melanoma are rare lesions. NFs have to be differentiated from these spindle cell lesions. Presence of mast cells and fine fibrillar collagen matrix in NFs will differentiate it from schwannoma. A positive history of trauma for traumatic neuroma is essential for the diagnosis of this reactive lesion. Immunohistochemical positive reaction for cytokeratin and HMB-45 is necessary for the diagnosis of spindle cell carcinoma and amelanotic melanoma, respectively, which are negative for NFs.

## 4. Conclusion

Solitary NF is a benign neoplasm which occurs more commonly in extraoral sites. An intraosseous solitary NF of the jaw is very unusual and whenever an intraosseous, slow growing radiopaque mass in the jaws is observed, a neurofibroma can be included in the clinical differential diagnosis along with other common lesions like ossifying fibromas and osteomas.
